# Comparison of Mid-Upper-Arm Circumference and Weight-For-Height *Z*-Score in Identifying Severe Acute Malnutrition among Children Aged 6–59 Months in South Gondar Zone, Ethiopia

**DOI:** 10.1155/2021/8830494

**Published:** 2021-05-05

**Authors:** Dereje Birhanu Abitew, Alemayehu Worku Yalew, Afework Mulugeta Bezabih, Alessandra N. Bazzano

**Affiliations:** ^1^School of Public Health, Addis Ababa University, Addis Ababa, Ethiopia; ^2^School of Public Health, Bahir Dar University, Bahir Dar, Ethiopia; ^3^School of Public Health, Mekelle University, Mekelle, Ethiopia; ^4^School of Public Health and Tropical Medicine, Tulane University, New Orleans, LA, USA

## Abstract

Children with severe acute malnutrition (SAM) are identified for admission to outpatient therapeutic programs using mid-upper-arm circumference (MUAC) or weight for height (WHZ). However, MUAC and WHZ do not identify the same children, and such observed differences might have programmatic implications of missed nutrition therapy if only MUAC is used to identify children with SAM. The objective of the study was to assess any difference in prevalence and degree of agreement between MUAC and WHZ in identifying SAM affected children. A cross-sectional study was conducted in South Gondar Zone, Ethiopia, among 17 districts, with 3 districts and 10 health centers with their clustered health posts selected randomly. A total of 2,040 children were recruited, and data were collected using a parent questionnaire then entered into EpiData and analyzed using SPSS v 20. A total of 1,980 respondents (97.1%) were interviewed, all of whom were female and rural residents. Children's mean age in months was 23.2 (SD ± 9.7), and 54% were male children. The prevalence of SAM based on MUAC <11.5 cm was 11.2% (95% CI: 9.9–12.7) and 11.0% (95% CI: 9.7–12.5) based on WHZ <−3. The agreement between MUAC and WHZ was good (*k* = 0.729). The proportion of children with SAM identified using both MUAC and WHZ was 61.2%. The prevalence of SAM identified using both MUAC and WHZ was comparable. A substantial degree of agreement between MUAC and WHZ was observed to diagnose SAM. Therefore, MUAC can be used as an appropriate tool in identifying children with SAM for admission into the outpatient therapeutic program (OTP) in the study area.

## 1. Introduction

Undernutrition is the result of nutrient deprivation either due to lack of adequate intake or repeated infection [1] and is categorized as chronic or acute malnutrition based on the duration of deprivation [2]. Acute malnutrition is further classified as moderate acute malnutrition (MAM) or severe acute malnutrition (SAM) based on the degree of malnutrition and the presence of edema [2]; therefore, it is SAM if weight-for-length/height *z*-score (WHZ) is below −3 SD and/or with MUAC <115 mm and/or with bilateral edema and moderately acutely malnourished (MAM) if WHZ is between −2 and −3 or MUAC between 115 and 125 mm [3, 4].

According to a 2018 report, globally over 49 million children under age five were wasted and nearly 17 million were severely wasted [5], while the magnitude nationally in Ethiopia and Amhara Region was found to be 7.2% and 7.6%, respectively [6]. Children with severe acute malnutrition have a high risk of death exceeding 9-fold compared to well-nourished children [7, 8]. Worldwide, about one million children die every year from SAM [9, 10], and in Ethiopia, about 57% of all under-five deaths are related to malnutrition, of which three-quarters result from complications associated with mild-to-moderate malnutrition [11].

Acutely malnourished children (with severe or moderate malnutrition) were traditionally identified using WHZ, particularly in primary healthcare settings, but measurement of height and weight are often challenging, especially for inexperienced health professionals and community healthcare workers, as this needs technical and practical skills; therefore, MUAC, a relatively simple procedure using simple colored plastic, was introduced as an alternative [8, 12, 13], and in the currently updated SAM management protocols, MUAC<115 mm or WHZ <−3SD are used as independent indicators for the identification of SAM [14].

Even if both WFH and MUAC can identify children with SAM [2], there are arguments about which may identify more SAM cases. Some studies have reported that a relatively similar magnitude of acutely malnourished children are identified if MUAC or WHZ is used [2, 15–20], while other studies reported that more SAM cases are identified if WHZ is used than MUAC [21, 22]. On the other hand, more SAM cases were reportedly detected if MUAC was used rather than WHZ [23].

Poor level of agreement between MUAC and WHZ in identifying SAM cases has also been reported ranging from 0 to 54% [2, 15, 16, 18, 22, 24–26]. Moreover, the discrepancy also varies in terms of child age, sex, and residential area [2, 27, 28].

Such observed differences might have programmatic implications in that depending on whether MUAC or WHZ is used in identification, and then acutely malnourished children could unreasonably miss nutrition therapy [2]. Therefore, the objective of this study was to assess differences in prevalence and degree of agreement between WHZ and MUAC in identifying SAM affected children aged 6 to 59 months in South Gondar Zone, Amhara Region, Ethiopia.

## 2. Methods and Materials

The full and complete methods for the study have been previously described in a publication related to “Predictors of relapse of acute malnutrition following exit from community-based management program in Amhara Region, northwest Ethiopia: An unmatched case-control study” [29] and in a previous publication related to risk of SAM following exit from care titled “Rural children remain more at risk of acute malnutrition following exit from community-based management of acute malnutrition program in South Gondar Zone, Amhara Region, Ethiopia: A comparative cross-sectional study” [30].

### 2.1. Study Design and Setting

This was a community-linked, facility-based cross-sectional study conducted from 10 November 2017 to 30 January 2018 in the South Gondar Zone of Amhara Region, Ethiopia. The zone has 17 districts, five of which are town administrations. Debretabor is the capital city of the zone situated about 100 Km east of Bahir Dar (the capital city of Amhara Region) and 667 km north of Addis Ababa (the capital city of Ethiopia). According to the Government of Ethiopia, the 2017/18 population of the zone was 2,484,929 of which 183, 525 were children 0–4 yrs old [31]. For more detail of the study area and setting, see previous references [29, 30].

### 2.2. Population and Inclusion/Exclusion Criteria

The sampling frame for this study was children aged 6–59 months who had been discharged as recovered from the community-based management of acute malnutrition (CMAM) program and age-matched children who had never been treated for SAM in South Gondar Zone, Amhara Region, Ethiopia. The study population constituted children aged 6–59 months in the randomly selected districts of South Gondar Zone. Children aged 6–59 months in randomly selected districts were included, while children with chronic illnesses or congenital malformations or with visible spinal deformity were excluded. Additional details of how the study population was selected, and the detailed inclusion and exclusion criteria, can be found in previous publications [29, 30].

### 2.3. Sample Size Determination and the Sampling Procedure

The sample size for this study was 2,040 based on the calculation for the two objectives of the project: 1,318 sample size taking 95% CL, 16.9% relapse of SAM [32], 3% margin of error, design effect of 2, and 10% nonresponse rate,and for the other objective, taking the percent of acute malnutrition among recovered children following exit from CMAM, 78% [33], with an assumption of a 10% difference in the children age 6–59 months who were never treated for SAM, with 95% CL, 80% power, a ratio of 1 : 1, design effect of 2, and including 10% nonresponse rate, the final sample size for the comparison group was 722, and therefore, the final sample size was 2,040. The outcome variable was severe acute malnutrition (yes vs. no), while the independent variables were demographic, socioeconomic, household hygiene/sanitation, awareness of recommended caring and feeding practices for children, health facility access, and household food security status. Regarding the sampling procedure, a two-stage sampling technique was used. Among the 17 districts, 3 rural administrative districts (Ebnat, Tach-Gayint, and Lay-Gayint) were selected, within which 10 health centers with their clustered health posts were selected. Finally, through these identification procedures, mothers/caretakers of children were contacted by HEWs and asked to bring the children for assessment. The details of the sample size calculation and the sampling procedure can also be found in previous publications [29, 30].

## 3. Operational Definitions

### 3.1. Severe Acute Malnutrition (SAM)

A child was considered as severely acutely malnourished if he/she had MUAC <11.5 cm or WHZ <−3 and/or had bilateral edema [2].

### 3.2. Safe Child Feces Disposal

Child feces disposal was considered safe if the child useda latrine or child feces was rinsed into a latrine and considered unsafe otherwise.

### 3.3. Improved Water Source

A household was considered to have improved drinking water if the source was either from the pipe, protected spring, protected well, and/or boiled water.

### 3.4. Good Hand Washing Practice

A respondent was categorized as having good hand washing practice if they reported washing hands at 3 or more critical times/points (before eating/feeding a child, before preparing food/cooking, after defecation, and after cleansing the child's bottom) [34].

### 3.5. Household Food Insecurity Status

Food security was determined using the 9-item Household Food Insecurity Access (HFIA) scale questions. Before assigning the food insecurity category (access), each frequency of occurrence responses was coded as 0 for all cases where the answer to the corresponding occurrence question was “no,” and then, the four food security categories were computed and created sequentially into the HFIA category 1 as food secure, category 2 as mildly food insecure, category 3 as moderately food insecure, and category 4 as severely food insecure according to FANTA recommendation [35]. Finally, HFIA category 1 was considered as food secure and the remaining as food insecure.

### 3.6. Data Collection Tools and Measurements

The data collection tools used consisted of a checklist and a questionnaire that were prepared using the therapeutic program (SC/OTP) multichart which is utilized throughout the country [36]. Additionally, the questionnaire used for this study was adapted from validated, locally used questionnaires in nutrition research and survey reports such as the Ethiopian Demographic and Health Survey report [37]. Moreover, questions to assess HH's food security status were taken from a validated questionnaire developed by the Food and Nutrition Technical Assistant (FANTA) project [35].

Mothers were interviewed using the questionnaire which took approximately 20–30 minutes. The data collection took place from 10 November 2017 to 30 January 2018. A total of 15 data collectors were recruited and trained for 2 years, with training content mainly focusing on anthropometric measurement techniques [2, 36]. The data collectors were closely supervised by the 3 trained health professionals and by the principal investigator.

### 3.7. Anthropometric Measurements

A wooden measuring board was used for measuring length/height which was measured in recumbent and/or standing position based on their age and was recorded to the nearest 0.1 cm. Children's weight was also taken using a salter/beam balance scale based on their age. Child MUAC was measured using a nonstretchable tape according to the standard and recorded to the nearest 0.1 cm. The presence of edema was assessed and recorded according to accepted standards [2, 36]. The details of the data collection tools, how they were validated, how the measurements were taken, the operational definitions used, and how data quality was assured can be found in [29, 30].

### 3.8. Data Management Analysis Ethical Issues

The questionnaire was checked manually for completeness and was entered into EpiData version 3.3.2 and exported to SPSS version 20 for analysis. Indices were generated according to the WHO 2006 Child Growth Standards [38] using WHO Anthro software 3.2.2. Descriptive statistics such as mean and standard deviation (SD) were computed for continuous and percentages for categorical variables. The agreement between MUAC and WHZ in identifying acutely malnourished children was estimated by computing weighted kappa. The protocol and consent form were approved by the institutional review board (IRB) of the College of Health Sciences of Addis Ababa University, with an IRB protocol number of 068/16/SPH and meeting number of 001/2917. Written permission letters were obtained from the Regional Health Bureau, South Gondar Zone Health department, and at each District Health Offices of Amhara Region, Ethiopia. Informed verbal consent was obtained from all study respondents after the purpose, risk, benefit, confidentiality, and degree of involvement were fully explained to parents/caregivers in their local language, and children with MAUC <11.0 cm or presence of edema were linked to OTP as this was the currently used admission criterion to therapeutic care.

## 4. Results

### 4.1. Background Characteristics

Overall, there were 2,040 respondents, of which, 1,980 were interviewed (97.1% response rate). All respondents were female gender, rural residents, and Amhara ethnicity. The mean age in years of respondents was 29.1 (SD ± 6.6), and the majority (87.0%) were in the age range of 20–39 years. The mean age of children in months was 23.2± (SD 9.7) and about half (49%) were in the age range of 12–23 months, and 54% were male children. The majority (87%) were farmers by occupation, 82% had no formal education, and 75% were food-secure households (see Table 1).

### 4.2. The Prevalence of Acute Malnutrition Based on MUAC and WHZ

**Figure 1:**
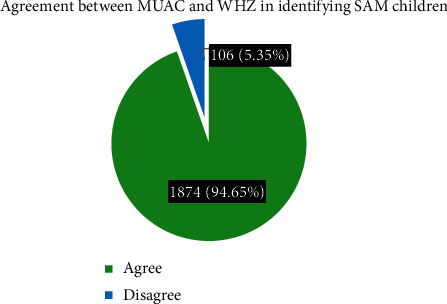
A figure showing frequencies and the corresponding proportions for agreement and disagreement between MUAC vs. WHZ in identifying SAM children following recovery from CMAM, South Gondar Zone, Amhara Region, 017/18 (*n* = 1980).

The prevalence of SAM was 11.2% (95% CI: 9.9–12.7) based on MUAC < 11.5 cm and 11.0% (95% CI: 9.7–12.5) based on WHZ < −3. The proportion of SAM children identified based on MUAC <11.0 cm was 6.1% (95% CI: 5.1–7.3) and based on either MUAC <11.5 cm or WHZ < −3 was 13.8% (95% CI: 12.3–15.4). SAM was more common among males than female children using both indicators, but no significant difference was observed in the child age. The overall prevalence of acute malnutrition was 32.0% (95% CI: 30.4–34.1) based on MUAC <12.5 cm and 27.3% (95% CI: 25.3–29.3) based on WHZ <−2, as illustrated in Tables2 and 3, Figure 1.

### 4.3. Agreement between MUAC and WHZ in Identifying SAM Children

A weighted Kappa (Cohen's) test was calculated using a 2 by 2 table to determine the level of agreement between MUAC <11.5 and WHZ <−3 in identifying SAM children. The overall agreement between MUAC and WHZ was good (*K* = 0.729). The proportion of SAM children identified using both MUAC and WHZ was 61.2%. See Table 4 for further illustration.

## 5. Discussion

Currently, WHO guidelines recommend the use of low mid-upper-arm circumference (MUAC <115 mm), low weight-for-height (WFH < −3 *z*-scores of WHO standards), and/or edema as internationally recognized independent diagnostic criteria for severe acute malnutrition in children age 6–59 months [2, 14] and at community-based programs; however, it is recommended to use only MUAC and edema as criteria to admit children with SAM to the OTP [8].

In the current study, the ability of the two indicators (MUAC <11.5 cm and WHZ < −3) to identify SAM children was compared, and the findings indicated that the proportion of SAM affected children identified by the two indicators (MUAC <11.5 cm and WHZ < −3) were comparable, while a lower proportion of affected children were identified based on the admission criteria (MUAC <11.0 cm) used at the data collection time compared with the recommended criteria (MUAC <11.5 cm and WHZ <−3) [14]. The finding supports the WHO and UNICEF 2009 report where the prevalence of SAM based on MUAC <11.5 cm and WHZ <−3 was very similar [2]. Similar findings were reported among Nigerian children with SAM [16]. A systematic review has also reported that MUAC performed at least as well as measures of W/H to identify SAM children [39]. A study in Southern Ethiopia indicated a nonsignificant difference in the prevalence of SAM based on MUAC and WHZ [23]. However, a study in Pakistan identified more children with SAM by MUAC compared to WHZ [20]. Moreover, two studies in Niger reported that more cases were identified using MUAC than WHZ [24, 40]. But, a study in Sudan indicated that more SAM cases were identified using WHZ than with MUAC [21]. A study in South Africa also reported the identification of more children with SAM with WHZ than using MUAC [15].

When considering the prevalence of global acute malnutrition (GAM) (MUAC<12.5 cm or WHZ <−2), MUAC identified more acutely malnourished children compared with WHZ. The finding was in agreement with a study conducted in Niger that more acutely malnourished children were identified using MUAC than WHZ [40]. Also, a study in Southern Ethiopia described that MUAC categorized more children as wasted compared with WHZ [23]. On the other hand, a study in South Africa reported W/H to be more sensitive than MUAC to identify acutely malnourished children [15]. Moreover, a study in Somalia revealed that GAM was higher based on WHZ than based on MUAC [41].

The proportion of acute malnutrition (SAM or MAM) identified based on MUAC and WHZ has also been reported by analyzing anonymous data from 1,832 anthropometric surveys from 47 countries indicating that only a minority of children were diagnosed as malnourished using these criteria. Moreover, the magnitude and direction of discrepancy varied dramatically between countries, with some having most children diagnosed malnourished by MUAC and in others by WHZ alone [25].

The variation in the prevalence of acute malnutrition (both SAM and MAM) based on these two indicators (MUAC and WHZ) could, therefore, be associated with different aspects such as body composition. This may be because WHZ is more influenced by body shape than MUAC. For example, a study in Ethiopia considering children from agrarian and pastoralist areas identified that agrarian children were having higher sitting to standing ratio of height (SSR) values than pastoralist children. Therefore, WHZ may not be more affected by SSR in agrarian than in pastoralist (long-legged) children. Thus, WHZ and MUAC yielded similar estimates in agrarian but different estimates in pastoralist children [17]. A nonsignificant but high proportion of SAM children both using MUAC and WHZ and a high proportion of GAM based on MUAC compared with WHZ, therefore, may be associated with the agrarian nature of the study area. The variation in the prevalence of acute malnutrition may also be due to children's stunting status; a study has shown that stunted children tend to accumulate more fat mass and gain less lean body mass than nonstunted children and, therefore, perhaps, leading to lower MUAC cutoffs [42]. The higher stunting prevalence (41.3%) in the Amhara Region [6] and in the current study area (69%) could, therefore, have an effect on the prevalence estimate of acute malnutrition using these two indicators.

To examine the strength of agreement between MUAC and WHZ in identifying SAM children, Cohen's kappa (k) was used. The agreement between MUAC <11.0 cm and WHZ < −3 in identifying SAM children was marginally poor (*K* = 0.600), and the proportion of SAM children identified by both indicators was below half (46.1%), whereas the degree of agreement between the WHO-recommended MUAC cutoffs (MUAC <11.5 cm) and WHZ<−3 was substantial (*K* = 0.729) and a higher proportion of SAM children were identified by both indicators (61.2%). This may indicate that admission cutoff used at the data collection time (MUAC <11.0 cm) has been missed in identifying more SAM children than the WHO recommendation to change from MUAC <11.0 cm to MUAC <11.5 cm to increase diagnostic accuracy [43].

The agreement based on MUAC <11.5 cm was comparable to a study conducted in Southern Ethiopia with 71% agreement between MUAC <115 mm and WHZ <−3 [44]. Other studies also reported agreement in the two indicators. A study in the rural Gambia reported a 59.8% overlap between WHZ and MUAC in identifying SAM children [45]. The WHO and UNICEF report in 2009 indicated a 40% agreement in identifying SAM children using WHZ and MUAC [2], and in Niger, 39% agreement in SAM identification was reported [24]. On the other hand, a poor level of agreement between MUAC and WHZ was reported in Cambodia that SAM screening using MUAC<115 mm would have missed over 90% of children with WHZ <−3; conversely, WHZ <−3 missed 80% of children with MUAC <115 mm [27]. Furthermore, a study in Nigeria also reported that none of the children classified as SAM by WHZ were classified as SAM by MUAC [16]. Despite discrepancies among study findings, the admission criteria at data collection time could miss more SAM children, while the current to-be-used community-based screening using MUAC <11.5 cm to admit to OTP program is less likely to miss SAM children if only MUAC <11.5 cm is used as screening/admission criteria in the study area.

The WHO and UNICEF report in 2009 indicated that children with WHZ below −3 SD based on WHO standards have a high risk of death exceeding 9-fold compared with children with WHZ >−3 [7]. Similarly, children with MUAC below 115 mm showed an increased risk of dying [8] with a specificity of more than 99% in children aged 6–59 months in the two indicators [2]. A study in India indicated that MUAC predicted death better (sensitivity: 95.5%, specificity: 25.0%) than WHZ (sensitivity: 86.4%, specificity: 21.4%) [46]. In the current study, children were admitted to OTP based on MUAC <11.0 cm and/or edema, and sensitivity and specificity of MUAC <11.0 cm against WHZ <−3 in identifying children with SAM was 49% and 99%, respectively, but sensitivity is 77% and specificity is 97% if MUAC <11.5 cm was used as admission criteria. This may indicate that the risk of mortality associated with SAM would have been reduced if MUAC <11.5 cm was used as a screening and admission criteria than MUAC <11.0 cm.

Scholars even recommend MUAC cutoffs to be more than 11.5 cm to identify more SAM cases and, therefore, reduce mortality. A study in India indicated that the sensitivity and specificity of MUAC <11.5 cm against WHZ <−3 was 13.6% and 99.3%, respectively, recommending to increase the cutoffs to <12.8, so 50% sensitivity and 90.8% specificity [47]. Another study in the Wardha district of India also reported 23.5% sensitivity and 99.7% specificity for MUAC <11.5 cm and recommending cutoff to be MUAC<12.8 cm to diagnose SAM at 74.1% sensitivity and 93.2% specificity [48].

Regarding the sex of children, the prevalence of SAM in boys was higher compared with girls both using MUAC and WHZ. However, a study in South Sudan, the Philippines, Chad, and Bangladesh reported that male children were diagnosed more acutely malnourished either by WHZ <−2 or MUAC <125 but female children were detected as more malnourished by MUAC <125 only [49]. But a study in India indicated that MUAC <115 mm preferentially selected more girls than boys [50]. A study in southern Ethiopia also showed the prevalence of SAM in boys to be higher than girls when WHZ was used but identified no sex difference when MUAC was used [23].

When considering global acute malnutrition based on MUAC <12.5 cm and WHZ <−2, the prevalence was still higher in boys than girls, however this was not consistent with other research findings. For example, a study in Cambodia reported that WHZ identifies more acutely malnourished boys than girls while more females than male children using MUAC [28]. A study in Somalia also revealed that boys to be diagnosed as acutely malnourished by WHZ more than female children while girls were more acutely malnourished than boys using MUAC [41]. Therefore, it may not always be possible to state that MUAC is better at identifying acutely malnourished children more than WHZ, and vice versa.

The high prevalence of acute malnutrition (both SAM and MAM) among male compared with female children could be due to the coexistence of stunting. It was reported that acute malnutrition is linked to an increased risk for stunting, so this could limit optimal linear growth due to the occurrence of both wasting and stunting over several months in an individual child [51]. A study in rural Senegal to describe the patterns of concurrent wasting and stunting (WaSt) among children aged 6–59 months and found that WaSt was more highly prevalent (more stunting if wasted, more wasting if stunted) in boys than girls [52]. Descriptive epidemiology of multiple anthropometric deficits using data from 51 countries also indicated that WaSt is more common among male than female children [53], and also, in the current study, male children were found more wasted and stunted than female children (34.3% vs. 25.8%) and were more severely wasted and stunted than female children (15.3% vs. 10.2%). In the Ethiopian DHS 2019 report, male children were also found more stunted and wasted than female children [6], indicating that male children are more affected than female children in terms of these two indicators in the study area.

Despite the standardization of anthropometric instruments, intensive training, and close supervision, misclassification of children's nutritional status due to measurement error is potentially a limitation. There may also be age misclassification, as children's age relied on the respondent's recall. However, the use of multistage sampling to select study districts and allocating the sample size to the health posts proportionately, and the use of a clear definition of the outcome of interest and adequate sample size determined following a statistical method could allow findings of the current study to be generalized to the target population (children aged 6–59 months).

In conclusion, the proportion of children with SAM identified using both MUAC <11.5 and WHZ <−3 was comparable and a substantial degree of agreement was observed between MUAC and WHZ to diagnose severe acute malnutrition. MUAC has also shown to be more sensitive and specific in identifying SAM affected children. This may have important implications for CMAM programming in that use of only MUAC can still be an appropriate tool in identifying SAM in children for admission into the outpatient therapeutic program in the study area.

## Figures and Tables

**Table 1:** Background characteristics of respondents and children aged 6–59 months, South Gondar Zone, Amhara Region, Ethiopia, 2017/18 (*n* = 1980).

Variable	Response category	# (%)
Respondent age in yrs.	Mean ± SD	29.1 ± 6.6
	15–19	77 (3.9)
	20–29	916 (46.3)
	30–39	805 (40.7)
	40+	182 (9.2)

Child age in months	Mean ± SD	23.2± (9.7)
	6–11	120 (6.1)
	12–23	968 (48.9)
	24–35	627 (31.7)
	36–47	182 (89.2)
	48–60	83 (4.2)

Child sex	Male	1063 (53.7)
	Female	917 (46.3)

Respondent education status	No formal	1616 (81.6)
	Formal	364 (18.4)

Respondent occupation	Farming	1730 (87.4)
	Other than farming	250 (12.6)

HH family size	≥5	1088 (54.9)
	<5	892 (45.1)

HH food insecure	No	1481 (74.8
	Yes	499 (25.2)

Water source improved	Yes	1083 (54.7)
	No	897 (45.3)

Latrine (pit with or without cover)	Yes	1714 (86.6)
	No	266 (13.4)

Hand washing practice	Poor	1077 (54.4)
	Good	903 (45.6)

Child feces disposal (safe)	Yes	1127 (56.9)
	No	853 (43.1)

BF initiation within 1 hr of birth (*n* = 1977)	Yes	1283 (64.9)
	No	694 (35.1)

The practice of prelacteal feeding	Yes	107 (5.4)
	No	1873 (94.6)

Vitamin A supplementation in the past 6 months	Yes	1393 (70.4)
	No	587 (29.6)

History of illness in the past 2 wks.	Yes	346 (17.5)
	No	1634 (82.5)

Preparing food separately for children	Yes	1150 (58.1)
	No	830 (41.9)

Another family member on SAM treatment	Yes	42 (2.1)
	No	1938 (97.9)

**Table 2:** Some of the epidemiologic variables from the two parent studies [29, 30].

Variable	Response	Following recovery (*n* = 1273)	Comparison group of reference 30 (*n* = 707)
		Frequency (%)	Frequency (%)
Child sex	Male	680 (53.4)	383(54.2)
	Female	593 (46.6)	324 (45.8

Child age in month	6–11	50 (3.9)	70 (9.9)
	12–23	689 (54.1)	279 (39.5)
	24–35	387 (30.4)	240 (33.9)
	36–47	109 (8.6)	73 (10.3
	48–59	38 (3.0)	45 (6.4)

Respondent education status	Unable to read and write	768 (60.3)	404 (57.1)
	Read and write only	292 (22.9)	152 (21.5)
	Primary and above	213 (16.7)	151 (21.4)

Respondent occupation	Farming	1188 (93.3)	542 (76.7)
	Other than farming	85 (6.7)	165 (23.3)

HH family size	<5	558 (43.8)	334 (47.2)
	≥5	715 (56.2)	373 52.8()

HH food security status	Insecure	321 (25.2)	178 (25.2)
	Secure	952 (74.8)	529 (74.8)

Vaccinated for measles	No	58 (4.6)	105 (14.9)
	Yes	1215 (95.4)	602 (85.1)

Vitamin A supplemented in the past 6 months	No	318 (25.0)	269 (38.0%)
	Yes	955 (75.0)	438 (62.0)

Water source for drinking improved	No	598 (47.0)	299 (42.3)
	Yes	675 (53.0)	408 (57.7)

Type of latrine	Pit	1105 (86.8)	609 (86.1)
	Open field/bush	168 (13.2)	98 (13.9)

Hand washing practice	Poor	584 (45.9)	319 (45.1)
	Good	689 (54.1)	388 (54.9)

Safe child feces disposal practice	No	570 (44.8)	283 (40.0)
	Yes	703 (55.2)	424 (60.0)

HH own farmland	No	77 (6.0)	106 (15.0)
	Yes	1196 (94.0)	601 (85.0)

HH own animals	No	95 (7.5)	117 (16.5)
	Yes	1178 (92.5)	590 (83.5)

**Table 3:** Magnitude of acute malnutrition based on MUAC in cm and WHZ among children, South Gondar Zone, Ethiopia, 2017/18 (*n* = 1980).

		Severe acute malnutrition (SAM)				Global acute malnutrition (GAM)	
		MUAC <11.5	WHZ < −3	MUAC<11.5 and WHZ < −3	MUAC<11.5 or WHZ < −3	MUAC <12.5	WHZ < −2
Age in month		% (95% CI)	% (95% CI)	% (95% CI)	% (95% CI)	% (95% CI)	% (95% CI)
6–23	Combined (1088)	11.5 (9.6–13.4)	9.9 (8.2–11.9)	7.8 (6.3–9.6)	13.5 (11.5–15.7)	33.5 (30.7–36.4)	26.9 (24.3–29.7)
	Male (559)	14.1 (11.4–17.3)	13.8 (11.0–16.9)	11.7 (8.3–13.6)	17.2 (14.1–20.6)	39.5 (35.5–43.7)	32.2(28.3–36.2)
	Female (529)	8.5 (6.3–11.2)	5.9 (4.0–8.2)	4.7 (3.1–6.9)	9.6 (7.3–12.3)	27.2 (23.5–31.2)	21.4 (17.9–25.1)

24–59	Combined (892)	11.0 (9.0–13.2)	12.3 (10.2–14.7)	9.2 (7.4–11.3)	14.1 (11.9–16.6)	30.2 (27.2–33.3)	27.7 (24.8–30.8)
	Male (504)	12.3 (9.6–15.5)	14.3 (11.3–17.8)	10.5(8.0–13.5)	16.1 (13.0–19.6)	31.7 (27.7–36.0)	29.0 (25.0–33.1)
	Female (388)	9.3 (6.6–12.6)	9.8 (7.0–13.2)	7.5 (5.1–10.6)	11.6 (8.6–15.2)	28.1 (23.7–32.8)	26.0 (21.7–30.7)

6–59	Male (1063)	13.3 (11.3–15.5)	14.0 (12.0–16.3)	10.6 (8.8–12.6)	16.7 (14.5–19.0)	35.8 (33.0–38.8)	30.7(27.9–33.5)
	Female (917)	8.8 (7.1–10.9)	7.5 (5.9–9.4)	5.9(4.5–7.6)	10.5 (8.6–12.6)	27.6 (24.7–30.6)	23.3 (20.6–26.2)
	Total (1980)	11.2 (9.9–12.7)	11.0 (9.7–12.5)	8.4 (7.2–9.7)	13.8 (12.3–15.4)	32.0 (30.0–34.1)	27.3 (25.3–29.3)

**Table 4:** Agreement between MUAC and WFH in *Z*-score in identifying SAM children by age and sex, South Gondar, Amhara Region, Ethiopia, 2017/18 (*n* = 1980).

		SAM (WHZ < −3)	Not SAM (≥−3)	Total	
All children					
SAM (MUAC <11.5)		167	55	222	
Not SAM (MUAC ≥11.5		51	1707	1758	
Total		218	1762	1980	
			*K* = 0.729		

kap MUAC_Kappa WHZ_Kappa, wgt (w)					
Ratings weighted by:					
1.0000	0.0000				
0.0000	1.0000				

Agreement (%)	Expected agreement (%)	Kappa	Std. Err.	*Z*	Prob > *Z*
94.65	80.25	0.7290	0.0225	32.44	0.0000

## Data Availability

The data used to support the findings of this study are available from the corresponding author upon request.

## Abbreviations

CMAM: Community-based management of acute malnutrition
DHS: Demographic health survey
FANTA: Food and nutrition technical assistance
GAM: Global acute malnutrition
HEW: Health extension worker
HFA: Height for age
HFIAS: Household food insecurity access score
HP: Health post
IRB: Institutional review board
MAM: Moderate acute malnutrition
MUAC: Mid-upper-arm circumference
OTP: Outpatient therapeutic program
SAM: Severe acute malnutrition
SC: Stabilization centre
SSR: Sitting to standing ratio
UNICEF: United Nations Children's Fund
WaSt: Wasting and stunting
WFA: Weight for age
WFH: Weight for height
WHO: World Health Organization.
